# Elderly Consumers’ Risk of Accidental Subscription in Micro-Drama Platforms: A Demographic and Behavioral Analysis

**DOI:** 10.3390/bs16060929

**Published:** 2026-06-05

**Authors:** Yarnaphat Shaengchart, Pongsakorn Limna, Kanchana Viriyapant, Nalinpat Bhumpenpein

**Affiliations:** 1 International College, Pathumthani University, Mueang 12000, Pathum Thani, Thailand; 2 Faculty of Information Technology and Digital Innovation, King Mongkut’s University of Technology North Bangkok, Bangsue, Bangkok 10800, Thailand; s.yarnaphat@email.kmutnb.ac.th (Y.S.); nalinpat.b@itd.kmutnb.ac.th (N.B.)

**Keywords:** accidental subscription, elderly consumers, micro-drama platforms, digital consumer behavior, demographic factors, behavioral factors

## Abstract

This study examines the risk of accidental subscription among elderly consumers in micro-drama platforms, addressing a critical gap in digital consumer behavior research as aging populations increasingly engage with subscription-based digital services. Using a quantitative approach, data were collected from 780 Thai respondents aged 60 and above through a structured online questionnaire. The data were analyzed using binary logistic regression to assess the effects of demographic factors (age, gender, education, and income) and behavioral factors (platform usage frequency, time spent per session, prior subscription experience, and impulse clicking behavior) on the likelihood of accidental subscription. The findings reveal that age, gender, platform usage frequency, time spent per session, and prior subscription experience significantly influence accidental subscription, while education, income, and impulse clicking behavior do not. Notably, frequent platform use and prior experience increase risk, whereas longer session duration reduces it, suggesting nuanced engagement effects. These results confirm that accidental subscription is a systematic and predictable outcome shaped by user characteristics and interaction patterns. The study contributes by extending consumer behavior research to unintended outcomes and offers practical implications for user-centered platform design, consumer protection policies, and targeted digital literacy initiatives, particularly in emerging digital economies.

## 1. Introduction

The rapid advancement of digital technologies has significantly transformed consumer behavior, particularly in the domain of digital entertainment and platform-based services. The proliferation of mobile applications, short-form video platforms, and subscription-based content has reshaped how users consume media, emphasizing immediacy, personalization, and continuous engagement (Filieri et al., 2025; Roussel, 2025; Cui & Mohib, 2025). Micro-drama platforms—featuring short, serialized video content optimized for mobile viewing—have rapidly emerged as a key segment of the digital entertainment ecosystem. These platforms rely on subscription and micro-transaction models, supported by interface designs that maximize engagement and monetization. Unlike traditional media formats, micro-dramas employ condensed narratives, strong visual hooks, and frequent cliffhangers to sustain attention in brief viewing contexts. Consequently, they represent both a narrative and industrial shift, shaped by platform economies, mobile consumption patterns, and algorithm-driven distribution (Z. Chen, 2025; Li et al., 2025; Pornsuppayakul et al., 2026; Zhou & Chen, 2025). Thailand provides a highly relevant context for examining these trends, as the country is simultaneously undergoing rapid digital transformation and demographic aging. As an emerging digital economy, Thailand has experienced significant growth in internet penetration, mobile usage, and digital platform adoption across all age groups. At the same time, the country has transitioned into an aging society, with individuals aged 60 years and above accounting for an increasing proportion of the population. Recent studies indicate that elderly users in Thailand are increasingly adopting digital technologies for communication, information access, and entertainment purposes, reflecting broader patterns of digital inclusion (Jantavongso, 2022; Sriwisathiyakun & Dhamanitayakul, 2022; Waiyawassana & Maen-in, 2023). According to the 2022 Thailand Internet User Behavior (IUB) survey, 96% of Baby Boomers (aged 58–76) use mobile phones and spend an average of 3 h and 21 min per day on the internet, compared with the national average of 7 h and 4 min. Internet usage is highest in Bangkok, averaging 10 h and 5 min daily, while users in the northeast, central, and northern regions report similar levels of 6 h and 59 min, 6 h and 45 min, and 6 h and 17 min, respectively. The southern region records the lowest usage at 5 h and 35 min per day (3.02%) (Pituk et al., 2025).

Despite these advancements, elderly consumers remain vulnerable to digital risks due to disparities in digital literacy and technological competence. Digital literacy is widely recognized as a critical factor influencing individuals’ ability to effectively navigate online environments and make informed decisions. Empirical evidence suggests that older adults often face challenges in understanding complex digital interfaces, recognizing online risks, and interpreting transactional information, which may increase their susceptibility to unintended actions (Aleti et al., 2025; Fan et al., 2026; Khan et al., 2026). In the Thai context, variations in digital literacy are strongly associated with demographic characteristics such as education level and income, thereby reinforcing inequalities in digital capability among elderly populations (Kaewmano, 2025; Pituk et al., 2025). These vulnerabilities are particularly significant in digital platforms that employ subscription-based monetization models. Many contemporary platforms incorporate features such as auto-renewal subscriptions, one-click payments, and time-limited offers, which are designed to streamline user experience but may also obscure financial commitments (L. Chen, 2023; Yang et al., 2025). Recent research has highlighted the role of persuasive and manipulative interface designs—often referred to as “dark patterns”—in influencing user behavior and increasing the likelihood of unintended purchases or subscriptions (Maier & Harr, 2020; Zac et al., 2025). Such design strategies can be especially problematic for elderly users, who may have limited prior experience with digital payment systems and reduced ability to critically evaluate interface cues (Kültür, 2025; Shapiro, 2025).

Given its growing relevance, it is imperative to examine elderly consumers’ susceptibility to accidental subscription within micro-drama platforms. Age-related factors—such as diminished cognitive processing speed, limited familiarity with rapidly evolving digital interfaces, and comparatively lower levels of digital and financial literacy—may substantially increase vulnerability to inadvertent subscription decisions. Elderly users, in particular, may encounter difficulties in interpreting interface cues, distinguishing between free and paid content, and navigating complex or persuasive design features embedded in subscription-based systems. These demographic constraints are further exacerbated by behavioral patterns, including repeated exposure, habitual viewing, and prolonged engagement, which collectively heighten the likelihood of unintentional financial commitments. As micro-drama platforms increasingly employ monetization strategies characterized by seamless, frictionless, and often subtly embedded subscription prompts, a nuanced understanding of how elderly consumers interact with such environments becomes critically important. This perspective not only advances theoretical insights into digital consumer behavior but also underscores the ethical imperative for more inclusive and responsible platform design.

Despite the extensive body of literature on digital consumer behavior, social commerce, and technology adoption, limited scholarly attention has been directed toward the phenomenon of accidental subscription, particularly among elderly populations in emerging digital entertainment contexts such as micro-drama platforms. Prior research has predominantly focused on intentional purchasing behavior, thereby overlooking unintended or inadvertent outcomes arising from the interaction between user characteristics and persuasive platform environments. To address this gap, this study investigates how demographic and behavioral factors influence accidental subscription among elderly consumers in Thailand, thereby providing empirical insights into digital consumer vulnerability in aging societies. Importantly, accidental subscription behavior should not be interpreted merely as an individual error or isolated purchasing mistake. Rather, it reflects the interaction between persuasive platform architectures and users’ varying levels of cognitive, behavioral, and digital vulnerability. In this context, demographic characteristics such as age, gender, education, and income are theoretically relevant as they shape individuals’ digital literacy, cognitive processing capacity, risk perception, and ability to navigate complex digital environments. Meanwhile, behavioral factors represent the more immediate mechanisms through which users engage with platform interfaces and subscription systems. Accordingly, this study conceptualizes accidental subscription as a structurally induced behavioral outcome emerging from the interaction between user vulnerability and persuasive digital design. The expected contributions of this study are both theoretical and practical. Theoretically, the study extends the digital consumer behavior literature by integrating demographic and behavioral perspectives within the frameworks of bounded rationality (de Clippel & Rozen, 2024; Hernandez & Ortega, 2019), behavioral economics (J. Chen, 2024; Hasan et al., 2025), and digital consumer vulnerability (Capone et al., 2026; Verma et al., 2026) to explain accidental subscription behavior among elderly users. Practically, the findings are expected to inform improvements in platform interface design, enhance transparency in subscription mechanisms, and support the development of targeted digital literacy initiatives and consumer protection policies for elderly populations in emerging economies.

## 2. Literature Review

This literature review comprehensively synthesizes prior research across four key dimensions: (1) micro-drama platforms and accidental subscription; (2) demographic factors and elderly vulnerability in digital contexts; (3) platform design and behavioral dynamics in accidental subscription; and (4) the theoretical foundations underpinning the study and the development of research hypotheses.

### 2.1. Micro-Drama and Accidental Subscription

Micro-drama has emerged as a rapidly growing format in digital entertainment, characterized by short, serialized video content designed for mobile-first consumption. These platforms typically employ episodic cliffhangers, emotionally engaging narratives, and frictionless viewing interfaces to maximize user retention and continuous engagement. Monetization strategies are often embedded seamlessly within the user journey, including freemium models, pay-per-episode access, and auto-renewing subscriptions. While such mechanisms enhance platform profitability, they also introduce behavioral and design-related risks, particularly when payment prompts are integrated into moments of heightened emotional engagement or cognitive immersion (Akbar et al., 2026; Z. Chen, 2025; Jiang, 2025; Li et al., 2025; Pornsuppayakul et al., 2026; Q. Wu et al., 2025).

In this context, micro-drama platforms frequently utilize dark patterns—such as pre-selected subscription options, limited-time prompts, obscured cancellation processes, preselected checkboxes, auto-included services, and overly sensitive “buy now” interfaces—that may nudge users toward unintended financial commitments (Dickinson, 2025). The episodic and immersive narrative structure further amplifies this effect by sustaining emotional arousal and encouraging rapid, low-deliberation decision-making, thereby increasing susceptibility to unintended transactions. From this perspective, unintended or accidental subscription can be understood as an outcome of manipulative design practices that exploit users’ limited attention and cognitive constraints within digitally mediated environments.

Importantly, accidental subscription should not be interpreted solely as a consequence of individual deficiencies, such as age-related cognitive decline or limited digital literacy. Research on dark patterns and persuasive interface design indicates that such UI/UX structures systematically exploit universal cognitive biases, heuristic processing, and automatic decision-making tendencies that operate across demographic groups (Hayati, 2024; Rossi et al., 2024; Zac et al., 2025). From a bounded rationality perspective, algorithm-driven platforms further encourage rapid decision-making, increasing susceptibility to cognitive biases regardless of users’ technological competence, as they shape constrained choice architectures that reinforce impulsive behavior (Heo, 2025). Collectively, accidental subscription should therefore be framed less as a problem of user weakness and more as a systemic outcome of persuasive platform design and monetization strategies. Within this context, elderly consumers may be considered a particularly vulnerable group situated within a broader structural environment shaped by platform architectures.

Extending this argument, the effectiveness of dark patterns may vary across commonly used proxies of consumer vulnerability—age, income, and education—highlighting how differences in resources, digital literacy, and cognitive capacity shape susceptibility to manipulative design practices. This heterogeneity is particularly evident among elderly users, who may experience greater difficulty navigating fast-paced digital environments and interpreting subtle interface cues (Zac et al., 2025). In this regard, older adults illustrate how accidental subscription extends beyond a purely technological issue to become a behavioral and demographic phenomenon shaped by the interaction between platform design, age-related cognitive factors, and individual user characteristics.

### 2.2. Demographic Factors and Elderly Vulnerability

Demographic characteristics may fundamentally shape users’ susceptibility to risks, such as phishing and accidental subscriptions, by influencing digital competence, cognitive capacity, and risk evaluation (Agagu & Omorogiuwa, 2025; Kavvadias & Kotsilieris, 2025). Regarding age, older adults face distinct challenges in online environments, including difficulties in verifying content credibility, navigating complex interfaces, and managing information overload. These challenges are partly linked to age-related cognitive changes that impair the ability to distinguish between accurate and misleading information, increasing reliance on heuristics and the likelihood of errors. The effect is further intensified by low digital literacy, which limits their capacity to recognize potential risks (Di & Wang, 2025; Ghenai et al., 2026; Yue et al., 2026). Gender differences also appear to influence susceptibility to online threats; prior research suggests that women tend to adopt more cautious online behaviors and may be less vulnerable to phishing emails, whereas men are generally more inclined to take risks in digital environments (Agagu & Omorogiuwa, 2025; Kavvadias & Kotsilieris, 2025).

Education and income moderate these vulnerabilities by shaping access to technology and digital capability. Higher education enhances critical evaluation and comprehension of transactional processes, while lower educational attainment limits the ability to detect misleading design. Similarly, higher income increases exposure and familiarity with digital systems but may reduce sensitivity to small transactions, whereas lower income constrains digital competence (Lal et al., 2025; Panetta et al., 2025; Vik et al., 2024). Elderly vulnerability stems from the combined effects of age-related cognitive decline, lower digital literacy, and limited familiarity with evolving digital interfaces, which collectively increase susceptibility to misleading cues and unintended online actions (Agagu & Omorogiuwa, 2025; Kavvadias & Kotsilieris, 2025; Mohammed et al., 2026; Pituk et al., 2025). Accordingly, this study conceptualizes demographic variables not merely as statistical controls but as substantive predictors of digital vulnerability. These demographic characteristics directly influence cognitive capability, digital literacy, risk perception, and interaction patterns in online environments, particularly among elderly populations who are disproportionately vulnerable to evolving platform designs and persuasive digital interfaces.

### 2.3. Platform Design and Behavioral Dynamics in Accidental Subscription

The platform economy transforms consumption into a continuous, data-driven process in which user decisions are actively shaped by algorithmic personalization and interface design (Correia, 2025; Naidoo & Hattingh, 2025). Behavioral finance literature indicates that repeated exposure to frictionless payment systems conditions consumers to prioritize convenience over careful evaluation, ultimately weakening long-term financial planning. Evidence from subscription-based ecosystems further suggests that concealed auto-debits and recurring micropayments can reduce spending visibility, contributing to a “silent wallet” effect in which financial outflows often go unnoticed. These dynamics are particularly pronounced in entertainment environments such as micro-drama platforms, where platform-driven viewing patterns foster high engagement, encouraging continued consumption while requiring subscription or payment to maintain access (Karthikeyan et al., 2025; Pornsuppayakul et al., 2026; Y. Wu, 2025; Zhao & Bian, 2025). Within such environments, persuasive design strategies—commonly conceptualized as dark patterns—systematically steer user behavior by exploiting cognitive biases, reducing transparency, and constraining alternative choices. Design tactics such as pre-selected options, hidden costs, and ambiguous confirmation processes embed financial commitments seamlessly into routine interactions, often without users’ full awareness. Repeated exposure to these interfaces reinforces habitual responses and automaticity, reflecting bounded rationality under conditions of information overload and time pressure (Dickinson, 2025; Nembaware & Sousa, 2025; Todewale et al., 2025; Zac et al., 2025).

Behavioral dynamics further shape how platform design translates into decision outcomes. Frequent platform usage increases exposure to transactional prompts and reduces perceived risk through familiarity; however, it also encourages automatic responses rather than conscious evaluation. Extended session duration may induce cognitive fatigue, impairing attention and increasing reliance on heuristics (Cho & Woo, 2025; Qin et al., 2024; Qiy, 2025). In addition, prior experience exerts a dual effect: while it can enhance procedural awareness, it may also foster overconfidence and reduced vigilance during routine interactions (Cheng et al., 2023; Kramer et al., 2022). Impulse clicking behavior further intensifies these risks, as users respond to salient cues with minimal cognitive processing (Malaviya et al., 2025, 2026). In this context, the convergence of engagement-driven design and behavioral tendencies shifts decision-making away from deliberate evaluation toward design-induced responses, thereby significantly increasing the likelihood of accidental subscription.

### 2.4. Theoretical Foundations and Hypothesis Development

This study is grounded in an integration of bounded rationality theory, behavioral economics, and digital consumer vulnerability frameworks, which collectively explain how individuals make decisions under conditions of limited cognitive capacity, information asymmetry, and persuasive digital environments. These perspectives are particularly relevant in digital platform contexts, where user decisions are shaped not only by individual characteristics but also by interface design and algorithmic structures. Within micro-drama platforms, the convergence of immersive content and monetization mechanisms creates an environment in which users are more likely to rely on heuristic processing, thereby increasing the likelihood of accidental subscription.

Bounded rationality suggests that individuals operate under cognitive limitations and often rely on simplified decision rules, especially in complex and fast-paced environments (de Clippel & Rozen, 2024; Hernandez & Ortega, 2019). Elderly consumers, in particular, experience age-related cognitive decline and lower levels of digital literacy, which constrain their ability to process information and recognize transactional risks (Di & Wang, 2025; Khan et al., 2026). Behavioral economics further explains how decision-making can be systematically influenced by contextual cues and design features (J. Chen, 2024; Hasan et al., 2025). Digital platforms frequently employ persuasive mechanisms, or dark patterns, such as default options, time-limited offers, and frictionless payment systems that exploit cognitive biases and reduce deliberate evaluation (Dickinson, 2025; Todewale et al., 2025; Zac et al., 2025). In addition, according to Capone et al. (2026) and Verma et al. (2026), the digital consumer vulnerability framework emphasizes that susceptibility to online risks varies across demographic and behavioral characteristics. Age, gender, education, and income influence digital competence and risk perception, while behavioral factors, such as usage frequency, shape exposure to persuasive design (Agagu & Omorogiuwa, 2025; Kavvadias & Kotsilieris, 2025; Qiy, 2025; Zac et al., 2025).

In this study, accidental subscription is conceptualized as a probabilistic outcome arising from the interaction between user vulnerability characteristics and platform-induced behavioral responses. Demographic variables capture structural differences in digital capability, cognitive processing, and online risk susceptibility, whereas behavioral variables reflect users’ direct interaction patterns with persuasive platform environments. Grounded in bounded rationality, behavioral economics, and digital consumer vulnerability frameworks, the following hypotheses are proposed:

Gender differences may influence online risk behavior and digital decision-making patterns. Prior research suggests that males tend to exhibit higher risk-taking tendencies, whereas females are generally more cautious in digital environments (Agagu & Omorogiuwa, 2025; Kavvadias & Kotsilieris, 2025). These differences may affect how elderly users evaluate subscription prompts and respond to persuasive platform interfaces. Accordingly, the following hypothesis is proposed:

**H1.**  *Gender (GEN) significantly influences the likelihood of accidental subscription among elderly consumers.*

Age is widely recognized as a key determinant of digital vulnerability. Older individuals often experience cognitive and perceptual limitations that reduce their ability to navigate complex digital interfaces, process transactional information, and identify misleading cues, thereby increasing susceptibility to unintended actions (Di & Wang, 2025; Yue et al., 2026). Accordingly, the following hypothesis is proposed:

**H2.**  *Age (AGE) has a significant positive effect on the likelihood of accidental subscription among elderly consumers.*

Education may influence digital vulnerability by enhancing individuals’ cognitive ability, critical thinking, and digital literacy, thereby improving their capacity to recognize manipulative design features and avoid unintended transactions (Lal et al., 2025; Panetta et al., 2025). Accordingly, the following hypothesis is proposed:

**H3.**  *Education level (EDU) has a significant negative effect on the likelihood of accidental subscription among elderly consumers.*

Income may shape digital behavior and online transaction patterns in multiple ways. Higher income may increase access to digital technologies and familiarity with online payment systems, while simultaneously reducing sensitivity to small recurring charges, potentially increasing the likelihood of unnoticed subscriptions (Panetta et al., 2025; Vik et al., 2024). Accordingly, the following hypothesis is proposed:

**H4.**  *Income level (INC) significantly influences the likelihood of accidental subscription among elderly consumers.*

Frequent platform usage increases exposure to subscription prompts and reinforces habitual interaction patterns. Repeated exposure may reduce perceived risk and encourage automatic responses rather than deliberate evaluation (Cho & Woo, 2025; Qiy, 2025). Accordingly, a hypothesis is proposed:

**H5.**  *Platform usage frequency (FRE) has a significant positive effect on the likelihood of accidental subscription.*

Time spent per session reflects the depth of engagement and potential cognitive fatigue. Extended exposure to digital content can impair attention and increase reliance on heuristics, thereby elevating the risk of unintended actions (Qin et al., 2024; Cho & Woo, 2025). Accordingly, a hypothesis is proposed:

**H6.**  *Time spent per session (TSP) has a significant effect on the likelihood of accidental subscription.*

Prior subscription experience can have both protective and adverse effects. While experience may improve procedural knowledge, it can also lead to overconfidence and reduced vigilance during routine interactions (Cheng et al., 2023; Kramer et al., 2022). Accordingly, a hypothesis is proposed:

**H7.**  *Prior subscription experience (PSE) has a significant effect on the likelihood of accidental subscription.*

Impulse clicking behavior reflects a tendency toward rapid, low-deliberation responses to digital stimuli. Such behavior is closely associated with heuristic processing and has been shown to increase vulnerability to unintended digital actions (Malaviya et al., 2025, 2026). Accordingly, a hypothesis is proposed:

**H8.**  *Impulse clicking behavior (ICB) has a significant positive effect on the likelihood of accidental subscription.*

Ultimately, the proposed hypotheses are theoretically grounded in established frameworks of bounded rationality, behavioral economics, and digital vulnerability. By integrating demographic and behavioral predictors, this study provides a comprehensive framework for explaining accidental subscription as an unintended outcome of human–technology interaction in persuasive digital environments.

## 3. Materials and Methods

This study adopted a quantitative research design to examine factors influencing Thai elderly consumers’ risk of accidental subscription on micro-drama platforms. A quantitative research design was adopted because the study aimed to examine the statistical relationships between demographic and behavioral factors and the likelihood of accidental subscription among elderly users. This approach enabled hypothesis testing, objective measurement of variables, and predictive analysis using binary logistic regression, thereby providing empirical and generalizable findings.

Data were collected using a structured online questionnaire administered through Google Forms as the primary research instrument. The measurement items were developed through a comprehensive review of the relevant literature on digital consumer behavior, elderly digital vulnerability, and accidental subscription behavior, and were adapted from established and validated scales where appropriate (Aleti et al., 2025; L. Chen, 2023; J. Chen, 2024; Kraiwanit et al., 2023; Kumar & Patel, 2025). The study’s questionnaire consisted of four sections. Section 1 collected demographic information. Section 2 examined digital platform usage behavior. Section 3 measured behavioral tendencies related to accidental subscription, particularly impulse clicking behavior and interaction patterns on micro-drama platforms. Behavioral and usage-related items in Section 2 and Section 3 were measured using a five-point Likert scale ranging from 1 = strongly disagree to 5 = strongly agree. Section 4 assessed accidental subscription experience, which served as the dependent variable and was operationalized as a binary outcome indicating whether respondents had previously experienced accidental subscription on micro-drama platforms (1 = Yes, 0 = No).

The draft questionnaire was evaluated by three experts in elderly consumer research, demographic and behavioral studies, and survey methodology. Based on their feedback, items such as “I often click on promotional content while using short-video platforms” were revised to reduce ambiguity and improve contextual relevance for older users. The Item Objective Congruence (IOC) values ranged from 0.80 to 1.00, indicating strong content validity and alignment with the study objectives. A pilot test with 30 participants was then conducted, during which respondents reported difficulties with terms such as “subscription trigger” and “auto-renewal behavior.” Accordingly, terminology was simplified (e.g., replacing “subscription trigger” with “unintended subscription event”), question order was revised to move general usage behavior before sensitive behavioral items, and overlapping items were removed to reduce redundancy, thereby improving reliability and validity. To accommodate varying levels of digital literacy among elderly respondents, the questionnaire used simplified wording, large and readable formatting, and clearly structured response categories (e.g., separating “Yes/No” items from Likert-scale items). For instance, platform-related terms such as “micro-drama app interface” were briefly explained as “short video entertainment applications such as TikTok-style platforms.” Where necessary, additional explanations were provided to ensure consistent understanding and improve response accuracy.

A convenience sampling approach was employed, supplemented by snowball sampling in which initial respondents were encouraged to share the survey link within their social and family networks. These non-probability techniques were appropriate given the difficulty of accessing elderly individuals with varying levels of digital literacy through conventional online recruitment. They also enabled broader participation across different levels of technological familiarity while remaining feasible within the study context. Nevertheless, the study acknowledges that non-probability sampling may limit representativeness and generalizability, and this limitation is recognized accordingly.

To ensure participant eligibility and data reliability, screening questions were included at the beginning of the questionnaire to verify that respondents were aged 60 years or above, resided in Thailand, and had prior experience using digital platforms, particularly micro-drama or short-video applications. Responses that did not meet these eligibility criteria were excluded from the final dataset.

Participants were recruited through multiple digital platforms, including Facebook and LINE, which are widely used communication platforms among older adults in Thailand. To improve recruitment transparency, respondents were specifically approached through elderly-focused Facebook groups, LINE community networks, senior citizen associations, community-based online groups, personal contacts, and referrals from initial respondents. These channels were selected because they are commonly used by Thai older adults for communication, social interaction, and information sharing (Kleechaya, 2021; Kraiwanit et al., 2023).

Because some elderly participants experienced difficulties with smartphone use, internet navigation, or questionnaire access, family members, caregivers, or trained research assistants were permitted to provide limited technical assistance during survey completion. Technical assistance was strictly limited to procedural support, such as opening the survey link, scrolling through questions, adjusting device settings, or clarifying how to select responses. Assistants were explicitly instructed not to interpret questions, suggest answers, or influence respondents’ decisions in any manner. All responses ultimately reflected the participants’ own perceptions and decisions. Approximately 18% of respondents reported receiving limited technical assistance during survey completion, whereas the majority completed the questionnaire independently. Nevertheless, the study acknowledges that external assistance may introduce potential response bias despite the safeguards implemented.

Participation was voluntary and preceded by informed consent, with respondents assured that their data would be used solely for academic purposes. The target population comprised individuals aged 60 years and above in Thailand with prior experience using digital platforms, particularly micro-drama applications. The required minimum sample size for this study was determined using Cochran’s formula, applying a 95% confidence level and a 5% margin of error, which yielded a threshold of 384 participants (Uakarn et al., 2021). To improve precision and enhance the reliability of the findings, the study ultimately recruited 780 participants, ensuring adequate statistical power and stable parameter estimates. The online questionnaire was distributed over a three-month period, from January to March 2026. This duration was considered sufficient to obtain an adequate number of responses for the study.

To ensure methodological transparency and data integrity, all responses underwent systematic data screening and preprocessing prior to analysis. Incomplete questionnaires were excluded to maintain consistency across variables. Responses were also examined for straight-lining, extreme response patterns, duplicate entries, and unusually short completion times that may indicate inattentive answering. Outliers were assessed using standardized residuals and boxplot inspection to minimize the influence of extreme values on the results. Missing data were minimal and handled using listwise deletion. In addition, internal consistency and construct reliability were assessed prior to hypothesis testing, while coding and transformation procedures were applied where necessary to ensure comparability across variables. These procedures ensured that the dataset was robust, reliable, and suitable for subsequent statistical analysis.

Quantitative data were analyzed using descriptive and inferential statistics in Jamovi (version 2.16.17.0). Binary logistic regression analysis was employed to examine the influence of demographic and behavioral factors on the dependent variable, “accidental subscription on micro-drama platforms,” which was operationalized as a binary outcome. This approach enabled the estimation of the likelihood of accidental subscription among elderly users and generated odds ratios to determine the strength and direction of each predictor’s effect, thereby providing insights into the key factors contributing to accidental subscription behavior among Thai elderly consumers.

This study’s methodological design provides a rigorous framework for examining accidental subscription as a probabilistic outcome shaped by demographic and behavioral factors. By integrating appropriate sampling, participant verification procedures, validated measurement, and robust analytical techniques, the study enhances methodological transparency, internal validity, and practical relevance, contributing meaningfully to the literature on digital consumer behavior.

## 4. Results

The study collected comprehensive demographic and behavioral profile information from 780 respondents to contextualize the analysis of accidental subscription among elderly users in Thailand.

As presented in Table 1, the sample consisted of 52.3% male and 47.7% female, with an average age of 60.6 years, reflecting the targeted elderly population. In terms of education, 34.5% held a bachelor’s degree or higher, 41.8% completed secondary education, and 23.7% had primary education or below. Income was measured in monthly ranges, with 38.2% earning below THB 15,000, 44.6% earning between THB 15,001–30,000, and 17.2% earning above THB 30,000. Behavioral characteristics further captured users’ interaction patterns with digital platforms. A majority of respondents reported moderate to high platform usage frequency, with 62.7% accessing micro-drama platforms daily or several times per week. The average time spent per session was approximately 34 min, indicating substantial engagement exposure. Regarding prior subscription experience, 57.9% of respondents had previously subscribed to at least one digital service, suggesting varying levels of familiarity with subscription mechanisms. Notably, impulse clicking behavior was evident, with 48.6% of respondents reporting a tendency to click quickly without fully reviewing information. Collectively, these demographic and behavioral characteristics provide a robust and multidimensional profile of the sample, supporting a nuanced examination of how individual attributes and interaction patterns jointly influence the likelihood of accidental subscription.

Table 2 presents the Omnibus Tests of Model Coefficients, which assess whether the inclusion of predictor variables significantly improves the model compared to a baseline model with no independent variables. The results indicate that the model is statistically significant (χ^2^ = 211.844, df = 8, *p* < 0.001), confirming that the set of demographic and behavioral variables collectively contributes to predicting accidental subscription among elderly users. The significance of the step, block, and overall model demonstrates that at least one of the predictors has a meaningful effect on the dependent variable. This finding supports the suitability of proceeding with logistic regression analysis and indicates that the proposed model provides a significantly better fit than a null model.

Table 3 summarizes the overall explanatory power and goodness-of-fit of the logistic regression model. The −2 Log Likelihood value of 867.803 suggests an acceptable model fit, with lower values indicating better fit relative to alternative specifications. The Cox and Snell R^2^ (0.238) and Nagelkerke R^2^ (0.317) values indicate that the model explains approximately 23.8% to 31.7% of the variance in accidental subscription behavior. While these values suggest moderate explanatory power, they are consistent with expectations in behavioral research, where outcomes are influenced by multiple unobserved factors. The statistically significant chi-square value (χ^2^ = 108.935, *p* < 0.001) further confirms that the model provides a meaningful improvement over the baseline, reinforcing its adequacy for explaining variations in accidental subscription.

Table 4 reports the classification accuracy of the logistic regression model in predicting accidental subscription outcomes. The model correctly classifies 79.8% of non-subscription cases and 75.0% of subscription cases, resulting in an overall prediction accuracy of 77.3%. These results indicate a relatively strong predictive performance, demonstrating that the model is effective in distinguishing between individuals who are likely and unlikely to experience accidental subscription. The balanced classification rates across both categories further suggest that the model does not exhibit substantial bias toward either outcome, reinforcing its reliability for predictive purposes.

The predictive regression equation of Model 1 using the coefficients from Table 5 can be described by the following equation:

$$P=\frac{1}{1+e^{-z}}\;\text{-----------}\;Model\;1$$

where *P* is an accidental subscription among Thai elderly users, and *Z* = −2.489 − 0.930(GEN) + 0.574(AGE) + 1.133(FRE) − 1.537(TSP) + 0.915(PSE).

Table 5 presents the results of the binary logistic regression analysis, indicating the direction, magnitude, and statistical significance of each predictor of accidental subscription among Thai elderly users. Each coefficient (B) reflects the direction of the relationship, while the odds ratio (Exp(B)) indicates the change in the odds associated with a one-unit increase in the predictor.

Among the demographic factors, gender and age were found to significantly influence accidental subscription. Gender has a statistically significant effect (B = −0.930, *p* < 0.001; Exp(B) = 0.395), indicating that a one-unit increase in the gender variable (coded as male) decreases the odds of accidental subscription. This implies that male respondents are less likely to experience accidental subscription compared to female respondents, suggesting that gender-based differences in digital behavior and risk perception influence susceptibility to unintended subscription. Age shows a significant positive effect (B = 0.574, *p* < 0.001; Exp(B) = 1.775), indicating that a one-unit increase in age increases the odds of accidental subscription and confirms that higher age is associated with greater vulnerability. Education level is not statistically significant (B = 0.148, *p* = 0.347; Exp(B) = 1.160), indicating that variations in educational attainment do not meaningfully influence the likelihood of accidental subscription in this sample. This suggests that formal education alone may not adequately capture digital competence or the ability to navigate complex platform interfaces among elderly users. Income does not exhibit a significant effect (B = 0.075, *p* = 0.435; Exp(B) = 1.077). Although income may influence access to digital technologies, it does not appear to significantly affect the likelihood of accidental subscription in this context.

Regarding behavioral factors, platform usage frequency, time spent per session, and prior subscription experience significantly influence accidental subscription. Platform usage frequency has a significant positive effect (B = 1.133, *p* < 0.001; Exp(B) = 3.106), indicating that a one-unit increase in usage frequency increases the odds of accidental subscription and suggesting that repeated exposure to platform interfaces and subscription prompts reinforces habitual and automatic behaviors. Time spent per session shows a significant negative effect (B = −1.537, *p* < 0.001; Exp(B) = 0.215), indicating that a one-unit increase in time spent decreases the odds of accidental subscription and implying that extended engagement may allow users more time to process information and make more deliberate decisions. Prior subscription experience is also significant (B = 0.915, *p* < 0.001; Exp(B) = 2.496), indicating that a one-unit increase in prior experience increases the odds of accidental subscription and suggesting that familiarity may lead to overconfidence or reduced vigilance in routine interactions. Impulse clicking behavior is not statistically significant (B = 0.148, *p* = 0.509; Exp(B) = 1.159). Although theoretically relevant, this result indicates that impulsive tendencies do not significantly predict accidental subscription within this sample.

Table 6 presents the results of hypothesis testing based on the binary logistic regression analysis. The findings indicate that five of the eight proposed hypotheses were supported. Specifically, gender (H1), age (H2), platform usage frequency (H5), time spent per session (H6), and prior subscription experience (H7) were found to significantly influence the likelihood of accidental subscription among elderly consumers using micro-drama platforms. In contrast, education level (H3), income level (H4), and impulse clicking behavior (H8) did not demonstrate statistically significant effects and were therefore not supported.

Among the supported hypotheses, age, platform usage frequency, and prior subscription experience exhibited positive effects on accidental subscription, whereas time spent per session showed a significant negative effect. Gender also emerged as a significant predictor of accidental subscription. Overall, the results suggest that accidental subscription among elderly consumers is primarily influenced by demographic vulnerability and platform interaction behaviors rather than by socioeconomic characteristics or impulsive tendencies alone.

## 5. Discussion

This study examined the demographic and behavioral determinants of accidental subscription among elderly consumers using micro-drama platforms in Thailand. The findings provide important insights into how individual vulnerability characteristics and platform interaction patterns jointly shape unintended subscription behavior in digitally mediated environments. The results suggest that accidental subscription should not be interpreted merely as a random consumer mistake or isolated user error. Rather, it emerges as a probabilistic behavioral outcome influenced by the interaction between persuasive platform architectures and users’ varying levels of cognitive, behavioral, and digital vulnerability. The findings demonstrate that both demographic and behavioral factors significantly contribute to accidental subscription behavior. Among the demographic variables, age and gender emerged as significant predictors, whereas education and income did not exhibit statistically significant effects. From a behavioral perspective, platform usage frequency, time spent per session, and prior subscription experience significantly influenced accidental subscription, while impulse clicking behavior was not significant. Collectively, these findings highlight the importance of understanding accidental subscription not only through individual characteristics but also through the broader context of user–platform interaction and persuasive digital environments.

### 5.1. Theoretical Interpretation of the Findings

The findings can be interpreted through the lenses of bounded rationality, behavioral economics, and digital consumer vulnerability frameworks. Importantly, the results reinforce the argument that accidental subscription behavior is not simply the consequence of isolated user mistakes, but is structurally shaped by persuasive platform environments that encourage rapid, seamless, and low-deliberation decision-making processes. Within these theoretical perspectives, demographic variables are conceptualized not merely as descriptive characteristics or statistical controls, but as substantive indicators of digital literacy, cognitive processing capacity, online risk perception, and adaptive capability in complex digital environments. Consequently, factors such as age and gender reflect underlying differences in users’ ability to process transactional information, evaluate digital risks, and respond to persuasive interface designs. Accidental subscription emerges from the interaction between users’ demographic and behavioral vulnerabilities and platform architectures intentionally designed to promote frictionless engagement and automatic decision-making.

The significant positive effect of age supports bounded rationality theory, suggesting that cognitive limitations associated with aging—such as reduced processing speed, diminished attentional capacity, and lower adaptive capability in digital environments—constrain elderly users’ ability to critically evaluate complex subscription interfaces and transactional cues. In rapidly evolving digital ecosystems such as micro-drama platforms, users are frequently exposed to embedded subscription prompts, auto-renew mechanisms, and frictionless payment systems designed to minimize interruption and maximize conversion behavior. Under such conditions, older users may increasingly rely on heuristic processing and simplified decision-making strategies, thereby increasing susceptibility to unintended subscription outcomes.

The positive influence of age also supports digital consumer vulnerability perspectives, which emphasize that vulnerability emerges not solely from individual deficiencies but from the interaction between users’ cognitive constraints and persuasive digital architectures. Elderly users may experience greater cognitive overload when navigating fast-paced and highly immersive platform environments, particularly when transactional mechanisms are integrated seamlessly into entertainment experiences. Consequently, the findings suggest that accidental subscription among elderly consumers is partly a structural consequence of unequal adaptive capacity within increasingly complex digital marketplaces.

Gender differences further reinforce behavioral economics perspectives on digital decision-making and risk processing. The finding that male users are less likely to experience accidental subscription suggests that female users may be more influenced by emotionally immersive platform environments and interface-driven engagement mechanisms. Micro-drama platforms are intentionally designed to encourage continuous consumption through emotional storytelling, cliffhanger structures, and rapid interaction flows. Such persuasive environments may reduce deliberative evaluation and increase automatic responses to embedded payment prompts. This finding highlights the importance of considering how emotional engagement and interface design jointly shape consumer vulnerability within digital entertainment ecosystems.

Among the behavioral variables, platform usage frequency exhibited a strong positive effect on accidental subscription. This finding suggests that repeated exposure to subscription interfaces and monetization prompts fosters habitual behavior and automaticity. From a behavioral economics perspective, repeated interaction with similar interface structures may normalize transactional prompts and reduce users’ sensitivity to financial risk over time. Bounded rationality theory suggests that users repeatedly exposed to familiar interaction patterns may conserve cognitive effort by relying on routine behavioral scripts rather than actively reassessing each transactional decision. In persuasive digital environments where monetization mechanisms are embedded within continuous engagement loops, such habitual processing may substantially increase the likelihood of unintended subscription behavior.

Interestingly, time spent per session demonstrated a significant negative effect on accidental subscription. This finding suggests that longer engagement duration may facilitate more reflective and deliberate information processing rather than impulsive behavior. From a dual-process perspective, extended interaction time may allow elderly users to transition from rapid heuristic processing toward more analytical evaluation of payment conditions, subscription details, and transactional cues. Consequently, slower and more deliberate interaction patterns may partially mitigate the influence of persuasive platform design by encouraging users to process information more carefully before confirming payment-related actions. This finding highlights that not all forms of engagement necessarily increase vulnerability; rather, the quality and pace of engagement are critical determinants of digital decision-making outcomes.

Prior subscription experience also demonstrated a significant positive relationship with accidental subscription. Although prior experience is generally associated with increased competence and familiarity, the findings suggest a paradoxical effect in which familiarity may foster overconfidence and reduced vigilance. Consistent with behavioral economics theory, users who frequently engage with subscription systems may develop heuristic-based expectations that reduce careful verification behavior during routine interactions. Familiarity with digital payment systems may create a false sense of control, leading users to underestimate transactional risk and engage in automatic confirmation behaviors with limited cognitive scrutiny. This finding suggests that digital experience does not necessarily function as a protective factor; under frictionless and persuasive interface conditions, familiarity itself may become a source of vulnerability.

In contrast, education level and income did not significantly influence accidental subscription behavior. These findings suggest that formal socioeconomic indicators alone may not adequately capture users’ practical digital competence or susceptibility to persuasive platform environments. Although prior research often associates higher education with improved digital literacy and reduced online risk, the present findings imply that educational attainment may not necessarily translate into effective digital risk management within highly immersive and rapidly evolving digital ecosystems. Similarly, the non-significant effect of income suggests that accidental subscription behavior may be driven more strongly by interface design, behavioral exposure, and cognitive processing constraints than by financial resources. In low-cost microtransaction environments where subscription mechanisms are embedded seamlessly into entertainment experiences, financial considerations may become less salient during decision-making processes.

Impulse clicking behavior was also not statistically significant, despite theoretical expectations linking impulsivity with rapid and low-deliberation digital actions. This finding may indicate that, among elderly consumers, broader structural and contextual factors exert greater influence on accidental subscription than individual impulsive tendencies alone. Specifically, persuasive interface design, repeated exposure, cognitive overload, and familiarity effects may collectively outweigh the independent influence of impulsivity traits in shaping unintended subscription outcomes.

Collectively, the findings reinforce the argument that accidental subscription should not be viewed solely as the consequence of individual-level deficiencies or isolated consumer mistakes. Rather, it emerges through the interaction between human cognitive limitations and persuasive platform architectures intentionally designed to encourage rapid, frictionless, and low-deliberation decision-making. This shifts the analytical focus away from individual responsibility alone and toward the broader structural influence of digital platform design, monetization strategies, and dark pattern mechanisms in shaping consumer vulnerability among elderly users.

### 5.2. Comparison with Prior and Contradictory Evidence

A comparison of the findings reveals both alignment and divergence with prior literature when each significant and non-significant predictor is considered individually. Among the significant variables, age shows a positive effect on accidental subscription, consistent with prior research indicating that older adults experience cognitive decline, reduced information-processing capacity, and lower digital literacy, which increase their susceptibility to online risks (Di & Wang, 2025; Ghenai et al., 2026; Khan et al., 2026). This convergence reinforces the robustness of age as a key determinant of digital vulnerability. Gender is also a significant predictor, with males being less likely to experience accidental subscription. This finding is consistent with prior research indicating that gender differences influence online risk behavior and decision-making patterns (Agagu & Omorogiuwa, 2025; Kavvadias & Kotsilieris, 2025). Supporting this, Bai (2025) suggests that males generally exhibit stronger security awareness in digital contexts and tend to adopt more rational and analytical approaches to payment decisions. In contrast, females are more likely to rely on intuition and emotional experience when evaluating payment environments, partly due to habitual, rapid decision-making shaped by frequent online shopping. This reliance on intuitive processing, rather than systematic security evaluation, may increase sensitivity to interface design and, consequently, the likelihood of unintended subscription actions.

Turning to behavioral factors, platform usage frequency shows a strong positive effect, consistent with prior studies indicating that repeated exposure to digital platforms fosters habitual behavior and increases susceptibility to persuasive design and dark patterns (Cho & Woo, 2025; Qiy, 2025; Zac et al., 2025). Similarly, prior subscription experience is positively associated with accidental subscription, supporting earlier findings that familiarity may lead to overconfidence and reduced vigilance during routine digital interactions (Cheng et al., 2023; Kramer et al., 2022). This pattern is further supported by Bai (2025), who argues that individuals’ risk perceptions are shaped by psychological biases such as the familiarity effect, whereby repeated exposure to an environment reduces sensitivity to associated risks. In digital contexts, where users are often required to make rapid decisions under complex and information-constrained conditions, such reliance on prior experience and limited cues may increase the likelihood of unintended actions, including accidental subscription. However, time spent per session presents a contrasting result. While it is significant, its negative relationship with accidental subscription contradicts prior literature suggesting that prolonged digital engagement leads to cognitive fatigue and heuristic decision-making (Qin et al., 2024; Cho & Woo, 2025). Instead, the study’s findings suggest that longer session duration may facilitate more deliberate processing and careful evaluation, thereby reducing the likelihood of unintended actions.

In contrast, several non-significant variables diverge from established expectations. Education level does not significantly influence accidental subscription, contradicting prior research that identifies education as a key driver of digital literacy and risk mitigation (Lal et al., 2025; Panetta et al., 2025). However, this finding can be partially interpreted in light of Bai (2025), who reports that higher education may exert a paradoxical adverse effect in business transactions, suggesting a “curse of education” whereby highly educated individuals may become overconfident and exhibit reduced vigilance. This perspective implies that formal education does not necessarily translate into effective digital risk management. In the context of elderly users navigating rapidly evolving platforms, practical digital competence, familiarity with interface design, and experiential learning may be more critical than formal educational attainment in mitigating accidental subscription. Similarly, income is not a significant predictor, which contrasts with studies linking financial resources to digital access and behavior (Vik et al., 2024; Panetta et al., 2025). In microtransaction-based environments, the relatively low cost and recurring nature of subscriptions may reduce financial salience, thereby weakening the influence of income. Finally, impulse clicking behavior is not statistically significant, which diverges from prior findings that associate impulsivity with rapid, low-deliberation digital actions (Malaviya et al., 2025, 2026). This suggests that, within the elderly demographic, contextual and exposure-related factors may outweigh individual impulsivity traits in driving accidental subscription. Overall, these mixed findings highlight that while some predictors remain robust across contexts, others are highly dependent on user characteristics and platform-specific dynamics, underscoring the importance of contextualizing digital consumer behavior theories.

## 6. Conclusions

This study examined the determinants of accidental subscription among elderly consumers on micro-drama platforms in Thailand by integrating demographic and behavioral perspectives within a quantitative analytical framework. The findings demonstrate that accidental subscription is a systematic and predictable outcome shaped by specific user characteristics and interaction patterns rather than a purely incidental occurrence. Importantly, demographic variables in this study are not treated merely as descriptive characteristics or statistical control variables, but are theoretically grounded within the frameworks of bounded rationality, behavioral economics, and digital consumer vulnerability. In this context, demographic characteristics function as indicators of digital literacy, cognitive processing capacity, online risk perception, and adaptive capability within persuasive digital environments.

In particular, age and gender emerged as significant demographic predictors, while platform usage frequency, time spent per session, and prior subscription experience were identified as key behavioral determinants. The significant influence of age and gender suggests that differences in cognitive capability, heuristic processing, emotional engagement, and digital adaptation shape elderly consumers’ susceptibility to unintended subscription behavior. In contrast, education level, income, and impulse clicking behavior did not exhibit significant effects in this context, indicating that traditional socioeconomic indicators alone may not adequately capture users’ practical digital competence or vulnerability to persuasive interface design.

The results further highlight the critical role of exposure and experience in shaping digital vulnerability. Frequent platform usage and prior subscription experience increase the likelihood of accidental subscription, suggesting that repeated interaction with frictionless and persuasive interface designs fosters habitual, automated, and less deliberate decision-making processes. Conversely, longer time spent per session appears to reduce risk, indicating that deeper engagement may encourage more reflective evaluation of transactional cues and subscription conditions. These findings underscore the importance of distinguishing between different forms of digital engagement and their implications for consumer protection.

Ultimately, the study demonstrates that accidental subscription should not be viewed merely as an individual error or isolated consumer mistake, but rather as an outcome emerging from the interaction between users’ cognitive limitations, behavioral tendencies, demographic vulnerability, and persuasive platform design. As digital platforms continue to expand and monetize user engagement through increasingly seamless and embedded mechanisms, understanding unintended consumer outcomes becomes critically important. This study therefore provides empirical evidence and theoretical insights into the factors driving accidental subscription among elderly users, offering a foundation for future research and practical interventions aimed at fostering safer, more transparent, and more inclusive digital environments.

### 6.1. Research Contributions

This study makes several important contributions to the literature, particularly in the context of emerging digital economies. First, it extends digital consumer behavior research by shifting the focus from intentional transactions to unintended outcomes, thereby addressing a critical but underexplored aspect of platform-based consumption. By conceptualizing accidental subscription as a measurable and predictable phenomenon, the study broadens the analytical scope of consumer decision-making research. Second, the study contributes to the growing body of research on digital vulnerability among elderly populations in rapidly digitizing societies. In the Thai context, where digital adoption is increasing alongside demographic aging, the findings provide timely and contextually relevant insights. The identification of key predictors—such as age, gender, platform usage frequency, and prior subscription experience—offers a more nuanced understanding of how demographic characteristics shape users’ exposure and susceptibility to persuasive digital environments. Importantly, demographic variables are not treated merely as descriptive characteristics or statistical control variables; rather, they are theoretically grounded within the frameworks of bounded rationality, behavioral economics, and digital consumer vulnerability. In this study, demographic factors function as indicators of differences in digital literacy, cognitive processing capacity, online risk perception, and adaptive capability within rapidly evolving platform ecosystems. Accordingly, the findings demonstrate that digital inclusion does not necessarily equate to digital safety, particularly among elderly users who may face structural disadvantages when interacting with persuasive and frictionless subscription systems. Third, the study advances theoretical integration by combining demographic and behavioral perspectives within a single analytical framework. This integrated approach provides a more comprehensive explanation of accidental subscription by capturing both structural vulnerability characteristics and interaction-driven behavioral dynamics. In doing so, the study reinforces the argument that accidental subscription emerges through the interaction between users’ cognitive and behavioral constraints and persuasive platform architectures designed to encourage rapid and low-deliberation decision-making. Such an approach is particularly valuable in emerging contexts, where rapid technological change intersects with heterogeneous levels of digital capability and adaptive capacity. Finally, the findings have practical implications for platform design, policy development, and digital literacy initiatives. They underscore the need for more transparent subscription mechanisms, user-friendly interface design, and targeted interventions to protect vulnerable populations. These contributions are especially relevant for emerging economies, where regulatory frameworks, platform governance mechanisms, and consumer protection policies are still evolving.

### 6.2. Research Implications

The findings of this study offer important research and practical implications for platform developers, policymakers, and institutional administrators. First, the significant influence of age, platform usage frequency, and prior subscription experience suggests that accidental subscription is driven more by interaction patterns and cognitive constraints than by traditional socioeconomic factors. This indicates a need for future research to move beyond conventional predictors and incorporate more nuanced measures of digital behavior, interface complexity, and user–platform interaction. Practically, platform developers should implement user-centered design interventions, such as clearer subscription disclosures, mandatory confirmation steps for paid actions, simplified cancellation processes, and visual cues that distinguish free from paid content. Second, given that repeated exposure increases risk, adaptive interface mechanisms—such as usage-based warnings, spending alerts, or friction-increasing features for high-frequency users—should be explored and tested. For policymakers, the results support the development of stricter guidelines on dark patterns, including transparency requirements for auto-renewals and standardized consent protocols. Importantly, institutional administrators—such as those in universities, community centers, and public agencies—play a critical role in translating these insights into practice. They should design and implement targeted digital literacy programs for elderly populations, focusing on subscription awareness, interface navigation, and financial risk recognition. Additionally, administrators can collaborate with platform providers to co-develop training modules or awareness campaigns that simulate real-world scenarios of accidental subscription. Collectively, these implications not only guide future academic inquiry but also provide actionable strategies to reduce consumer vulnerability and promote safer digital engagement among aging populations.

### 6.3. Limitations and Future Research

Despite its contributions, this study is subject to several limitations that should be acknowledged. First, the use of convenience and snowball sampling may limit the generalizability of the findings. While the sample size is adequate for statistical analysis, it may not fully represent the broader population of elderly users in Thailand. Future research could employ probability sampling techniques to enhance representativeness. Second, the cross-sectional design of the study restricts the ability to infer causal relationships. Although the analysis identifies significant associations between predictors and accidental subscription, longitudinal studies are needed to examine how these relationships evolve over time and to establish causal mechanisms more robustly. Third, the study relies on self-reported data, which may be subject to recall bias and social desirability bias. Elderly respondents may have difficulty accurately recalling past behaviors or may underreport unintended actions. Future studies could incorporate behavioral tracking or experimental designs to obtain more objective measures of user behavior. Fourth, the operationalization of variables such as impulse clicking behavior and prior experience may not fully capture their complexity. More refined measurement scales or multidimensional constructs could provide deeper insights into these behavioral factors. Furthermore, future research may further refine the conceptual framework by treating demographic variables as contextual or control variables while placing greater emphasis on platform-specific and psychological constructs such as interface complexity, perceived transparency, digital literacy, and trust. Such an approach may provide a more focused theoretical explanation of how persuasive interface design, user engagement patterns, and digital interaction mechanisms shape unintended subscription outcomes among elderly consumers. In addition, future research could explore moderating or mediating variables, such as digital literacy, trust, interface complexity, and cultural factors, to develop a more comprehensive understanding of accidental subscription behavior. Comparative studies across countries or age groups may also provide valuable insights into the universality or context-specificity of the observed relationships.

## Figures and Tables

**Table 1:** Demographic Profile of Respondents (n = 780).

Variable	Category	Frequency (n)	Percentage (%)
Gender	Male	408	52.3
	Female	372	47.7
Age Group	60–64 years	356	45.6
	65–69 years	241	30.9
	70–74 years	121	15.5
	75 years and above	62	8.0
Education Level	Primary education or below	185	23.7
	Secondary education	326	41.8
	Bachelor’s degree or higher	269	34.5
Monthly Income	Below THB 15,000	298	38.2
	THB 15,001–30,000	348	44.6
	Above THB 30,000	134	17.2
Platform Usage Frequency	Daily or several times per week	489	62.7
	Occasionally	291	37.3
Prior Subscription Experience	Yes	452	57.9
	No	328	42.1
Impulse Clicking Behavior	Yes	379	48.6
	No	401	51.4
**Total**		780	100.0

**Table 2:** Omnibus Tests of Model Coefficients.

		Chi-Square	df	Sig.
Step 1	Step	211.844	8	<0.001
	Block	211.844	8	<0.001
	Model	211.844	8	<0.001

**Table 3:** Model Summary.

Step	−2 Log Likelihood	Cox & Snell R Square	Nagelkerke R Square	Chi-Square	Sig.
1 ^a^	867.803 ^a^	0.238	0.317	108.935	<0.001

^a^ Estimation terminated at iteration number 5 because parameter estimates changed by less than 0.001.

**Table 4:** Classification Table.

	Observed		Predicted		Percentage Correct
			Accidental Subscription in Micro-Drama Platforms		
			0	1	
Step 1 ^a^	Accidental Subscription in Micro-Drama Platforms	0	297	75	79.8
		1	102	306	75.0
	Overall Percentage				77.3

^a^ The cut value is 0.500.

**Table 5:** Variables in the Equation.

Variables		B	S.E.	Wald	df	Sig.	Exp(B)	95% C.I. for EXP(B)	
								Lower	Upper
Step 1 ^a^	GEN	−0.930	0.172	29.079	1	<0.001	0.395	0.282	0.553
	AGE	0.574	0.076	57.108	1	<0.001	1.775	1.530	2.060
	EDU	0.148	0.158	0.884	1	0.347	1.160	0.851	1.580
	INC	0.075	0.096	0.608	1	0.435	1.077	0.893	1.300
	FRE	1.133	0.212	28.525	1	<0.001	3.106	2.049	4.708
	TSP	−1.537	0.272	31.990	1	<0.001	0.215	0.126	0.366
	PSE	0.915	0.194	22.322	1	<0.001	2.496	1.708	3.649
	ICB	0.148	0.224	0.437	1	0.509	1.159	0.748	1.798
	Constant	−2.489	0.627	15.762	1	<0.001	0.083		

^a^ Variable(s) entered on step 1: gender (GEN), age (AGE), education level (EDU), income (INC), platform usage frequency (FRE), time spent per session (TSP), prior subscription experience (PSE), impulse clicking behavior (ICB). Note: Variables with *p*-values below 0.05 are accepted as significant predictors, while those with *p*-values above 0.05 are rejected as not statistically significant.

**Table 6:** Hypotheses Confirmation.

Hypothesis	Relationship	Result
H1	Gender → Accidental Subscription	Supported
H2	Age → Accidental Subscription	Supported
H3	Education → Accidental Subscription	Not Supported
H4	Income → Accidental Subscription	Not Supported
H5	Platform Usage Frequency → Accidental Subscription	Supported
H6	Time Spent per Session → Accidental Subscription	Supported
H7	Prior Subscription Experience → Accidental Subscription	Supported
H8	Impulse Clicking Behavior → Accidental Subscription	Not Supported

## Data Availability

The data supporting the findings of this study are available from the first or corresponding author upon reasonable request.

## Abbreviations

The following abbreviations are used in this manuscript:

IOC	Item Objective Congruence
GEN	Gender
AGE	Age
EDU	Education level
INC	Income
FRE	Platform usage frequency
TSP	Time spent per session
PSE	Prior subscription experience
ICB	Impulse clicking behavior
